# A rare clinical manifestation of cellular neurothekeoma in an elderly male patient

**DOI:** 10.1016/j.jdcr.2024.08.021

**Published:** 2024-09-02

**Authors:** Katherine Benandi, Devon Sieving, Catherine Campbell, Kristin Wolf, Trey Martin

**Affiliations:** a Complete Dermatology, Conroe, Texas; b John Sealy School of Medicine, University of Texas Medical Branch, Galveston, Texas; c Sagis Diagnostics, Houston, Texas

**Keywords:** cellular neurothekeoma, cutaneous tumor, soft tissue tumor, spindle cell lesion

## Introduction

Neurothekeomas (NTs) are superficial tumors with evolving diagnostic criteria. Previously, these tumors were classified as cellular, myxoid, or mixed-type based on the amount of myxoid matrix present.^1^, ^2^, ^3^, ^4^ The myxoid subtype is no longer considered to be a type of NT, because its neural origin and positive S-100 are more consistent with a diagnosis of nerve sheath myxoma. The NT diagnosis is now reserved for the cellular and mixed-type lesions, which are benign soft-tissue tumors commonly found on the head, neck, and shoulders. Clinically, they present as red, brown, or skin-colored papules or nodules and are typically painless.

NTs are most prevalent in young adult women, with a mean age of 15 to 21 years at presentation.^2^^,^^5^^,^^6^ Reports of these tumors are sparse in elderly patients, particularly men. An analysis of 178 NT tumors revealed a median age of 17 years, with the highest age of onset in an 85-year-old woman.^2^ In addition, a literature review of atypical cellular NTs (a rare variant of the cellular subtype) identified only 3 reported cases in patients aged >70 years, with the eldest man cited as 81 years.^7^ Other reports of NT in elderly patients include a cellular scalp lesion in an 88-year-old woman and a myxomatous lesion of the external ear canal in a 70-year-old man.^8^^,^^9^ The report below adds to this small body of existing literature describing NT cases in elderly male patients.

## Case report

**Fig 1:**
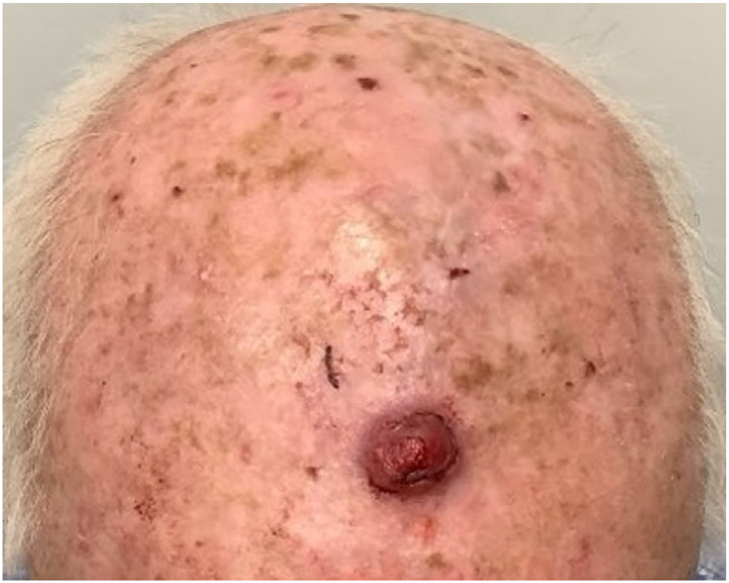
Neurothekeoma lesion on the scalp of an 86-year-old man at initial presentation.

An 86-year-old man with a medical history significant for prostate cancer presented with a 1.5 cm firm, dome-shaped, friable red-colored nodule on the frontal aspect of left side of his scalp (Fig 1).

**Fig 2:**
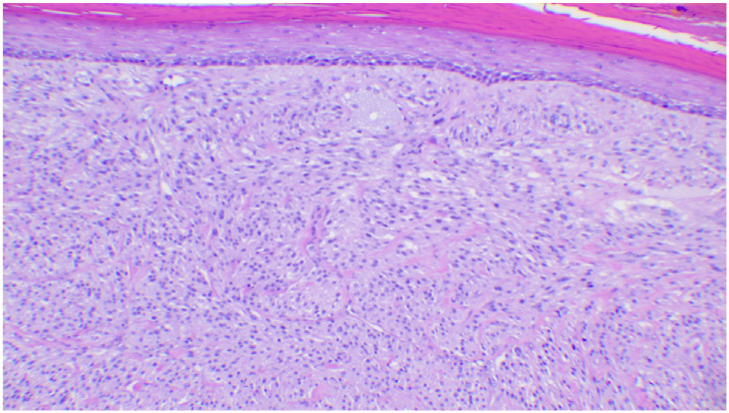
Hematoxylin-eosin stain showing a proliferation of spindled cells with increased mitotic figures. (Hematoxylin-eosin stain; original magnification: ×100).

**Fig 3:**
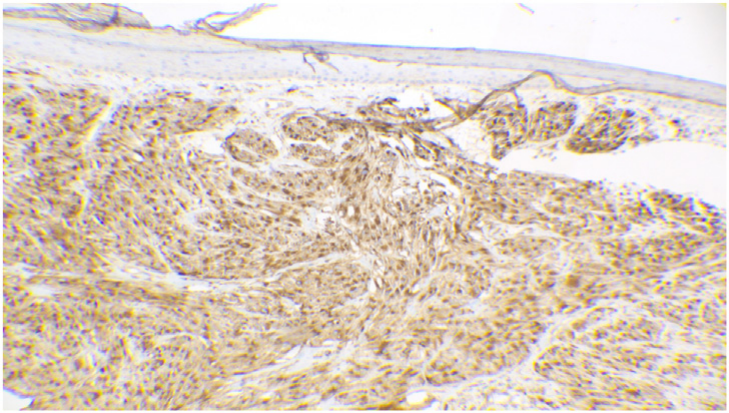
Immunohistochemical study with positive NKI-C3 staining. (NKI-C3 stain; original magnification: ×100).

Subsequent biopsy revealed a spindle cell lesion with increased mitotic figures and focal atypia with peripheral and deep margins involved (Fig 2). Overlying an effaced epidermis with a peripheral collarette, the lesion had neutrophilic and hemorrhagic crust. Epithelioid to spindled proliferation was present in the dermis with nest and fascicular arrangement. The nuclear features were bland, characterized by mild enlargement of nuclei containing scattered cells, small chromatin, small nucleoli, and relatively abundant eosinophilic cytoplasm. Cells stained positive for NKI-C3 (Fig 3) with increased proliferation rate of ∼10% in a Ki67/MART1 dual immunostain and negative for S-100, cytokeratin AE1/AE3, cytokeratin high molecular weight, SOX10, cytokeratin 8/18, CD117, epithelial membrane antigen, CD1a, CD34, actin, and anaplastic lymphoma kinase (D5F3) biomarker. Further, the preferentially expressed antigen in melanoma stain demonstrated partial immunoreactivity, whereas the CD68 and p16 stains demonstrated weak, nonspecific immunoreactivity. These findings were consistent with a diagnosis of cellular NT. The patient underwent complete excision of the lesion and is doing well with no evidence of recurrence.

## Discussion

We present a unique case of NT in an elderly male patient, with features contradicting the tumor’s classic manifestation in young female patients.^10^ Because of the clinical presentation and overlapping histologic features, atypical fibroxanthoma (AFX) was high on our differential diagnosis. The relative uniformity in cell size and shape in our patient’s lesion is notable on histology, a finding contrasting the marked pleomorphism of cells in AFX lesions.^11^ Additionally, AFX lesions classically contain multinucleated cells, which were not identified in our case.^11^ Further, cellular NT lesions are recognized by the formation of nests of epithelioid cells with swirling to fascicular growth pattern as observed in our case.^8^ Although AFX lesions may also demonstrate fascicular growth, they are more commonly associated with a haphazard or disorderly growth pattern and do not classically form nests.^11^ The lack of histologic features typically described in AFX lesions essentially excludes a diagnosis of AFX, despite clinical features classic for this lesion. Instead, the histologic findings are more in keeping with the diagnosis of a cellular NT lesion notable for its atypical clinical manifestation.

While examining demographic information in previously reported NT cases, the highest identified age for a male patient was 81 years old at onset. This case of NT presenting in an 86-year-old man surpasses this value by 5 years, thus widening the age range set by previous reports of this rare, benign tumor. Despite low incidence in this demographic, our findings should encourage providers to maintain some degree of clinical suspicion for this lesion in similarly presenting patients so they can be appropriately diagnosed and managed.

## Conflicts of interest

None disclosed.
